# Follow the heart or the head? The interactive influence model of emotion and cognition

**DOI:** 10.3389/fpsyg.2015.00573

**Published:** 2015-05-06

**Authors:** Jiayi Luo, Rongjun Yu

**Affiliations:** ^1^School of Psychology and Center for Studies of Psychological Application, South China Normal University, Guangzhou, China; ^2^School of Economics and Management and Scientific Laboratory of Economic Behaviors, South China Normal University, Guangzhou, China

**Keywords:** emotion, reason, dual-process, decision making, emotion regulation

## Abstract

The experience of emotion has a powerful influence on daily-life decision making. Following Plato’s description of emotion and reason as two horses pulling us in opposite directions, modern dual-system models of decision making endorse the antagonism between reason and emotion. Decision making is perceived as the competition between an emotion system that is automatic but prone to error and a reason system that is slow but rational. The reason system (in “the head”) reins in our impulses (from “the heart”) and overrides our snap judgments. However, from Darwin’s evolutionary perspective, emotion is adaptive, guiding us to make sound decisions in uncertainty. Here, drawing findings from behavioral economics and neuroeconomics, we provide a new model, labeled “The interactive influence model of emotion and cognition,” to elaborate the relationship of emotion and reason in decision making. Specifically, in our model, we identify factors that determine when emotions override reason and delineate the type of contexts in which emotions help or hurt decision making. We then illustrate how cognition modulates emotion and how they cooperate to affect decision making.

The heart has its reasons which reason does not know.

—Blaise Pascal

## Introduction

What is the distinction between emotion and reason? This question is as old as psychological science itself. Barrett (2012) suggested that emotions were both biologically evident and socially constructed. According to her conceptual act theory (Barrett, 2006, 2013; Barrett and Kensinger, 2010), physical states and actions can be transformed into different emotion expressions under different social contexts. Based on Barrett’s concept, we define emotion as a subjective, conscious experience characterized by biological reaction and mental states. When referring to reason, words like logic, analytic or reflective may come to mind. Reason plays a central role in our daily life, especially when we are confronted with different choices, decisions and judgments. According to classic rational decision theories (Kahneman and Tversky, 1979; Hey and Orme, 1994), we define reason as a process in which individuals analyze the pros and cons of the presented alternatives, calculate the utility of different options, and then choose one option that leads to a maximal profit.

The relation between reason and passion has fascinated philosophers for centuries. After Plato and Aristotle, western literature often treated reason as being opposed to emotion. This is the so called dilemma between “the head” (rationality) and “the heart” (emotion). Though, the exact relationship between reason and emotion remains a mystery, our daily experience leaves little doubt that both emotion and reason impact our decision making to a great extent. Decision making is often referred to as a process in which a choice is made after reflection about consequences of that choice. That is, making a decision requires knowledge about facts and values as well as involves the deliberation about consequence of the selected choice (Bechara and Van Der Linden, 2005). One example might help to illustrate the relationship of emotion and reason in decision making. Imagine that you are going to have a summer vacation and you have to choose between destinations A and B. During the decision making process, you firstly analyze the pros and cons of the two locations, and find that destination A is better than destination B. But at the same time, you remember a piece of recent news that there was an airliner crash in destination A. This news makes you anxious and you can anticipate the unpleasantness of the vacation assuming history repeats itself. You may also know that the chances of dying in a plane crash are extremely low and that flying is still incredibly more safe than driving. Eventually, you give up destination A and choose destination B instead. The contribution of both emotion and cognition is undeniable in this situation. However, the exact dynamic interplay between emotion and cognition remains to be fully explored. In this review, drawing on findings from behavioral economics and neuroeconomics, we firstly identify decision contexts in which emotion overrides reason to influence human behaviors and then discuss how cognition regulates emotion as well as how emotion and cognition cooperate to influence decisions. We then propose a novel model, labeled “The interactive influence model of emotion and cognition” (IIEC), to illustrate the relationship between emotion and reason. To note, because both the term “passion” and “emotion” represent humans’ subjective feeling and have been used interchangeably (Lazarus, 1996; Kappas, 2011a), then throughout the review we will employ the terms “emotion” and “passion” as well as the terms “reason” and “cognition” in an approximately interchangeable manner.

## When Does Emotion Dominate Cognition in Decision Making?

From an evolutionary point of view, emotion has evolved to guide behavioral responses in certain contexts. For example, immediate danger elicits fear, encouraging avoidance of close or looming threat (Mobbs et al., 2010); unfair treatment provokes anger, leading to more rejection even at great cost (Sanfey et al., 2003; Dawes et al., 2012). In this review, we propose that two typical contexts, labeled as “cognition reduction” and “emotion exaggeration,” may lead us to make a decision based more on emotion. In cognition reduction, cognitive capability is reduced so that it is less able to exert control over related tasks. Cognition reduction can be observed when information is incomplete (ambiguity), decision time is limited (time constraint), or self-control is impaired (ego depletion). In emotion exaggeration, emotional reaction is greatly enhanced, interfering with cognition and making a passionate response salient. This context can be seen when threat is proximal (proximity), stimuli are self-relevant (social distance), and equal resources are distributed unfairly (social instinct). From an evolutionary perspective, the context of emotion exaggeration creates a pressing need to react to environmental cues (Mobbs et al., 2010). In this section, we will identify the two contexts in which emotion overrides reason to influence decision making and try to explain the underlying mechanism within the reigning framework of dual-process models (Evans, 2008; Keren and Schul, 2009; Kahneman, 2011). Notably, the dual-process models suggest that mental processes are divided into two systems—one “hot” system that is emotional and intuitive, and another “cold” system that is rational and deliberative. The “cold” deliberative system battles against the “hot” intuitive (emotional) system (Seymour and Dolan, 2008). As intuitions are often in the form of emotional responses (Bechara et al., 1997; Monin et al., 2007; Helion and Pizarro, 2015), we then use intuitive process (in contrast to deliberative process) to represent emotional process in the following sections.

## Cognition Reduction: When Cognitive Capacity is Weak

Making a decision always requires cognitive resources and self-control. However, when cognitive capacity is weak, which we have labeled “cognition reduction,” emotion may overwhelm reason and become more salient in our decision making. In this section, we summarize evidence about how individuals rely more on emotion when cognitive capacity is insufficient in decision settings such as ambiguity, time constraint, and ego depletion.

### Ambiguity

Decisions are often required to be made when information is incomplete. Ambiguity refers to the situation where decision makers have no information about the mathematical probabilities of possible outcomes (Huettel et al., 2006). Recent neuroimaging evidence (Hsu et al., 2005; Huettel et al., 2006) suggests that ambiguity engages brain regions such as the orbital frontal cortex (OFC), the amygdala, and the dorsomedial prefrontal cortex (PFC). These regions have been implicated in reacting to emotional information (Critchley et al., 2000; Bechara et al., 2003) and integrating emotion and cognitive input (Critchley et al., 2001).

It seems that ambiguity elicits both a deliberation system (e.g., dorsomedial PFC) as well as an intuition system (e.g., amygdala), showing that reason and passion interplay to influence ongoing behaviors. However, we cannot conclude that emotion and reason are interactive in ambiguous situations because neural system responses depend on the degree of uncertainty. Relative to ambiguous conditions, situations involving clear risk (where the likelihood of different outcomes is expressed with certainty and mathematical precision) engage greater activation of the dorsal striatum (Hsu et al., 2005). The dorsal striatum is implicated in reward prediction (Knutson et al., 2001; O’Doherty et al., 2004), indicating that ambiguity decreases the anticipated reward associated with a decision. Moreover, on the behavioral level, consistent evidence shows that mean response time is faster in ambiguous gambles than in risky gambles (Huettel et al., 2006; Kuo et al., 2009). Taken together, the evidence from research in neuroeconomics suggests that ambiguous conditions engage the intuition system, allowing individuals to respond more rapidly and automatically. In contrast to the idea that ambiguity involves the emotional system, a meta-analytic study (Krain et al., 2006) showed that ambiguous decision-making elicited greater activation in the dorsolateral PFC relative to risky decision-making. These results indicate that ambiguous decision-making is associated with cognitive processes. The reasons for these different findings (ambiguous decision making is involved in cognitive processes or involved in emotional processes) are not yet clear, and require further investigation. Interestingly, when information is too complex or deliberation is too effortful, individuals also favor an intuitive process to help make sound decisions (Dijksterhuis et al., 2006). It is likely that deliberation leads to suboptimal weighting of unrelated information when issues are too complex, and therefore results in progressively worse choices (Dijksterhuis et al., 2006). Thus, we suggest that individuals favor an intuitive response when information is incomplete or when it is too complex.

### Time Constraint

**FIGURE 1:**
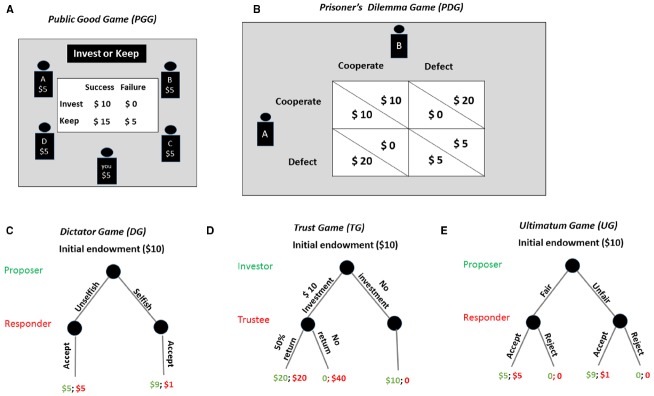
**Strategic decision making tasks. (A)** The public good game. In this task, subjects are given an amount of money, and they can decide how much to contribute to a common pool. The money contributed would be doubled and split evenly among the group members. **(B)** The prisoner’s dilemma game. The players each choose to either cooperate or defect with their opponent. Each player’s payoff depends on the interaction of the two choices. For example, the player A can get the largest payoff if he chooses to defect and player B chooses to cooperate, while the worst outcome occurs when he decides to cooperate but his partner defects. **(C)** The dictator game. The proposer determines a split of a certain endowment. The responder simply accepts the proposal. **(D)** The trust game. The investor decides whether or not to invest and how much of the endowment to invest with the trustee. The investment will be multiplied. The trustee is free to return any amount of the increased investment back to the investor; or he can keep all of the increased investment. **(E)** The ultimatum game. The proposer provides a proposal how to divide the money between the two while the responder has the option of accepting or rejecting the offer. If the responder accepts the offer, they both get the money as proposed; however, if the responder rejects the offer, neither player receives anything.

Researchers often study decision making in situations where decision makers have adequate time to perform the task. However, in real-life situations, decisions must often be made under time pressure. Time pressure is related to processing speed, which is a widely used psychological feature to distinguish intuition from reflection (Shiffrin and Schneider, 1977; Rand et al., 2012). In a recent study (Rand et al., 2012), investigators explored individuals’ cognitive mechanisms underlying cooperative behaviors by manipulating decision time. They assumed that if the individual was instinctively self-interested, then faster decisions would be less cooperative because prosocial decisions would require more deliberation to inhibit a selfish impulse; if the individual was instinctively cooperative, deliberation would lead to increased selfishness and faster decisions would be more cooperative. To test these competing hypotheses, they conducted a one-shot public good game (see Figure 1A) either with or without time constraints. Results showed a significantly higher contribution in the time pressure condition compared to the time delay condition, suggesting that faster decisions were associated with more prosociality (Rand and Nowak, 2013; Rand et al., 2014). Consistent findings are documented in other studies using the prisoner’s dilemma game (see Figure 1B; Dreber et al., 2008; Fudenberg et al., 2012) and the dictator game (see Figure 1C; Cornelissen et al., 2011).

The relationship between time constraint and cooperation is interpreted through the lens of evolutionary theory which suggests that intuitive prosociality is an outcome of cultural evolution and natural selection. Humans internalize cooperative strategies because they help us to better adapt in social interaction (Rand et al., 2012; Rand and Nowak, 2013). However, an evolutionary interpretation does not clarify the underlying mechanism by which time constraints affect decision making. Why the intuitive system dominates in the time pressure condition and how it works require further investigation. Although it remains unclear whether our innate responses are always prosocial, it is reasonable to believe that our responses under time pressure are the product of intuitive process rather than effortful reasoning.

### Ego Depletion

Cognitive resources and self-control are sometimes required during decision making. However, what if our cognitive resources are limited and our self-control is impaired? Ego depletion refers to a state in which acts of self-control draw on a resource that is limited, leading people to a depleted stage in which they are less able to exert self-regulation on a subsequent task (Baumeister et al., 1998; Hagger et al., 2010; Job et al., 2010). For example, subjects are forced to eat radishes instead of delicious chocolates leading to a subsequently faster quit on unsolvable puzzles than subjects who have not to exert cognitive control over eating (Baumeister et al., 1998). Individuals under ego depletion are more dishonest about their performance (Mead et al., 2009), less trustworthy in the trust game (see Figure 1D; Ainsworth et al., 2014), and more impulsive in making consumer choices (Baumeister, 2002) and other decisions (Bruyneel et al., 2009). A general explanation of the link between ego depletion and decision making is the limited resource model (Baumeister and Heatherton, 1996). This model indicates that there is a cognitive cost to resisting temptation; therefore, individuals under ego depletion do not have sufficient resources to override impulsive behavior (Bruyneel et al., 2009).

Though the limited resource model may help to explain the process of decision making under ego depletion, almost no data speak directly to the underlying mechanism of ego depletion’s effect on decision making. A recent study (Wagner and Heatherton, 2012) provided new insights into the process underlying ego depletion, demonstrating that self-regulatory depletion increased emotional reactivity in the amygdala and reduced functional connectivity between the amygdala and the ventromedial PFC. These findings suggest that ego depletion might disrupt the regulatory balance, such that regions engaged in emotional stimuli detection are amplified and the prefrontal regions engaged in top-down control are impaired. The results cannot be fully explained by the limited resource model, because increased emotional reactivity in the amygdala is observed but no reduction in activity in prefrontal regions is found. Thus, in this review, we speculate that if ego depletion works the same way in the decision making domain as in the emotion domain, then individuals’ performance in decision making may be due not to insufficient cognitive resources, but to dysregulation between cognition and impulses. Further research is needed to clarify how emotion and cognition fit together during self-regulation, using different scientific techniques such as functional neuroimaging and transcranial magnetic stimulation. These scientific techniques would help to directly explore the underlying mechanism of ego depletion and test the different theory demonstrating the effect.

## Emotion Exaggeration: When Emotion is Strong

Emotion plays a unique role in decision making (Green and Myerson, 2004; Seymour and Dolan, 2008). When emotion is greatly enhanced (e.g., induced by a proximal threat), it may interfere with cognitive function and exert a bottom-up influence on our decision making. In this part, we describe how humans’ tradeoffs are changed when emotion is exaggerated in decision settings such as proximity, social distance, and social instinct.

### Proximity

Emotion may have evolved to solve proximal problems that require immediate responses and influence our immediate survival. The proximal problem, the imminent threat, and even the immediate reward always push us to react rapidly. The animal model of fight–flight (Cannon, 1929) proposes that animals react to perceived threat with a discharge of the sympathetic nervous system—an autonomic nervous system that stimulates the body’s fighting and fleeing. More specifically, the adrenal medulla produces a hormonal cascade that results in the secretion of norepinephrine and epinephrine. This model also seems to apply to human beings when individuals confront something threatening. Imagine encountering a crawling spider nearby. Our instinctive defense reactions such as freeze and escape may be evoked for the sake of self-preservation. Using functional neuroimaging, researchers have begun to explore how the human brain responds to the proximity of threat (Mobbs et al., 2007, 2010). Data suggest that distal threat induces activity in the prefrontal cortices, reflecting the complex planning of avoidance strategies. When threat is proximal, midbrain structures (e.g., periaqueductal gray) become more active. Remarkably, the function of the amygdala is also well documented in fight–flight reactions to proximal threat (Mobbs et al., 2009; Volman et al., 2011a,b). In an approach-avoidance task (Volman et al., 2011a), inhibiting anterior PFC with continuous theta burst stimulation led individuals to commit more errors when they needed to select rule-driven responses (e.g., approach-angry) rather than automatic action tendencies (e.g., approach-happy) evoked by emotional faces. Concurrently, the inhibition of anterior PFC was accompanied by increasing activity in the amygdala during the approach-avoidance task. These findings show that imminent threat (e.g., angry emotional faces) results in autonomic and defensive reactions mediated by the amygdala. Taken together, the midbrain structures and the amygdala control reflexive escape behavior and fear-induced analgesia, suggesting that individuals are emotion dominated when facing proximal threat.

Emotional response to spatial distance can be extended to temporal distance (Kable and Glimcher, 2007). In an intertemporal choice task (Frederick et al., 2002; Green and Myerson, 2004), individuals are more impatient when choosing between an immediate reward and a delayed reward, than when choosing between a delayed and a more delayed reward. Neuroimaging research demonstrates that short-run impatience of this kind is mediated by the limbic system, which is implicated in reward processing (Breiter and Rosen, 1999). Notably, the limbic system has also been implicated in impulsive behavior and drug addiction (Bechara, 2005). For instance, individuals with drug addiction are over-responsive to drug-related cues, showing increasing activity in limbic components system such as the amygdala (Koob and Le Moal, 2007). It is possible that these affective signals can exert a bottom-up influence on the cognitive system. If the specific signals triggered by the emotion system are strong enough, they would have the capacity to override top-down cognitive control. In the proximal threat studies (Mobbs et al., 2007, 2010) and research on intertemporal choices (Kable and Glimcher, 2007), proximal threat and immediate reward seem to be these kinds of affective signals, which bias participants’ attention to proximal distance. Thus, both spatial distance and temporal distance modulate individuals’ reaction toward different stimuli. When stimuli are in proximal spatial distance and in the immediate time frame, individuals tend to switch from deliberative to intuitive processes and make instinctive responses.

### Social Distance

**FIGURE 2:**
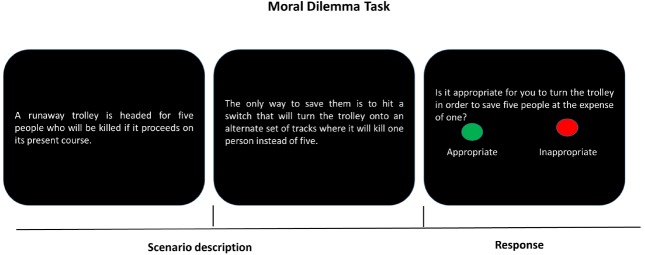
**A typical moral dilemma task.** The first two screens describe a scenario and the last screen poses a question about the appropriateness of an action in the specific scenario (e.g., turning the trolley). Participants respond by pressing one of two buttons (“appropriate” or “inappropriate”).

The term social distance refers to the widely accepted and consciously expressed norms about who should be considered an “insider” or an “outsider.” The norms, in other words, specify the distinctions between “self” and “others” (Akerlof, 1997; Bohnet and Frey, 1999). In social interaction, we are more emotionally involved in situations in which stimuli are self-relevant, but we are more analytic in situations where things are physically or psychologically distant from ourselves (Small and Loewenstein, 2003; Greene et al., 2004; Albrecht et al., 2010). For example, Albrecht et al. (2010) showed that when confronting a sooner-small and later-large reward, individuals chose more “sooner” options for themselves than for others. At the neural level, when choices included an immediate reward for self, brain regions including pregenual anterior cingulate cortex, ventral striatum, and anterior and posterior precuneus that were engaged in emotion and reward-related processing were activated; however, there were no neural activation differences between immediate and delay trials when individuals made choices for others. These findings suggest that choices for self and for others engage two distinct processes. Individuals hardly resist the temptation of meeting immediate gratification, while they focus more on long-term reward for others. This is in line with findings from research on moral judgment (see Figure 2). Personal moral judgment (e.g., pushing a stranger off a bridge onto the tracks to stop the trolley from hitting five people), as relative to impersonal moral judgment (e.g., turning the trolley away from five people but toward an alternative track on which stands one person), engages greater activity in brain areas associated with social-emotional processing, such as posterior cingulate gyrus and angular gyrus. In contrast, impersonal moral judgment, as relative to personal moral judgment, reveals a more prominent area of activation lying in the parietal lobe that is correlated with cognitive process (Greene et al., 2001, 2004).

Based on the aforementioned studies, we speculate that if making a choice for oneself and making a choice for others trigger different systems, then such distinct responses may generalize to other situations where there is no explicit but nevertheless an implicit “self” domain vs. “others” domain. Several studies support this hypothesis. For instance, subjects are more sympathetic with and generous toward specifically identified victims compared to “statistical” victims who are poorly identified, known as the “identifiable victim effect” (Small and Loewenstein, 2003; Kogut and Ritov, 2005). Moreover, when delving out punishment, people are more punitive toward identified wrongdoers than toward those who are unidentified (Small and Loewenstein, 2005). It is likely that vivid and concrete information is self-relevant to participants (Kogut and Ritov, 2005), leading to stronger emotional responses. Overall, social distance changes our decision making: the more self-relevant the situation is, the more emotions are involved when one makes decisions.

### Social Instincts

Emotion may function as a social signaling system. Anger tells others, “If you continue to provoke, I am ready to fight and attack.” This kind of anger implicates the inborn tendency (or drive) to react to the social interaction, and we define such a tendency and drive as social instincts. The ultimatum game (see Figure 1E; Güth et al., 1982) is widely used to study the association between social instincts (e.g., anger) and punishment. In this game, the proposer provides a proposal about how to divide the money while the responder has the option of accepting or rejecting the offer. If the responder accepts the offer, they both get the money as proposed; however, if the responder rejects the offer, neither player receives anything. An income-maximizing responder should accept any positive offer, and an income-maximizing proposer should offer the smallest possible amount to the responder (Xiao and Houser, 2005). However, studies using the ultimatum game demonstrate that when responders are offered 20% or so of the total amount, they are more likely choose to reject the proposal (Camerer, 2003). Even in advantage inequality situations (e.g., the responder receives a larger offer than the proposer in the ultimatum game), individuals dislike unequal outcomes and are willing to achieve a more equal reward distribution at their own personal financial cost (Tricomi et al., 2010; Yu et al., 2014).

Responders’ rejection and punishment in the ultimatum game may directly link to their feelings of anger and disgust (Pillutla and Murnighan, 1996; Sanfey et al., 2003; Lerner et al., 2004; Polman and Kim, 2013). Neuroimaging studies have observed that the insula and the dorsolateral PFC are activated in the contrast between “unfair—fair” offers (Sanfey et al., 2003; Dawes et al., 2012). Both anger and disgust have been seen to engage a distinct region of the anterior insula (Damasio et al., 2000). The higher the activation of the insula, the higher rate at which participants reject an unfair offer, indicating that activation of the insula may be associated with the degree of participants’ indignation and moral disgust in response to unfair offers (Sanfey et al., 2003; Dawes et al., 2012). By contrast, activation of the dorsolateral PFC suggests that individuals are trying to control their emotional impulses to reject unfair offers (Knoch et al., 2008; Tabibnia et al., 2008). During evolution, humans may have developed innate responses to emotionally charged situations such as being treated unfairly. Human brains might be hard-wired to react to certain social stimuli automatically and emotions such as disgust may facilitate such responses. Thus, social stimuli (e.g., unfairness) trigger emotions (e.g., disgust) that elicit impulsive responses (e.g., revenge).

### Critical Summary

In summary, we highlighted two contexts (cognition reduction and emotion exaggeration) in which cognitive capability was either reduced or interfered with by emotion-related cues or prior cognitive tasks. In the cognition reduction context, the cognition capacity is reduced by incomplete information (ambiguity), limited decision time (time constraint), and impaired self-regulation (ego depletion). In the emotion exaggeration context, emotional function is enhanced by affective signals that can exert bottom-up influence. These specific signals include threatening cues or immediate reward (proximity), self-related information (social distance), and social stimuli (social instinct). One common feature across the two contexts is that decision making engages greater activation in emotion-related brain regions such as the amygdala, the hypothalamus and the insula. Moreover, at the behavioral level, individuals behave more impulsively and make a faster choice in these two contexts. Because cognition fails to exert control in decision making in these contexts, passion becomes dominant and plays a central role in subsequent behaviors.

In this section, the competition between passion and reason can be interpreted within the framework of “dual-process models” (Evans, 2008; Keren and Schul, 2009; Kahneman, 2011). Dual-process models suggest that emotion is automatic, heuristic, and reflexive; whereas reason is controlled, analytic, and reflective (Evans, 2008). A great deal of research pursuing the notion of a dorsal-cognition vs. ventral-emotion axis organization of the human brain (Dolcos and McCarthy, 2006; Haas et al., 2006; Anticevic et al., 2010) suggests that emotion and reason are involved in two distinct mental modes. For example, Greene et al. (2001) demonstrated that when confronted with sacrificial moral dilemmas in which one individual must be sacrificed to save more people’s lives, participants often responded with an emotional reaction. Moreover, this gut reaction can be seen at the neural level that involving greater activation of emotion-related regions such as the posterior cingulate gyrus and the angular gyrus. However, when given enough time to analyze, individuals could shift from initial gut reactions to utilitarian responses. The researchers interpreted the results with the lens of divided mind—one system that produces an emotional judgment, and another deliberative system that can go against the intuitive initial reaction. The two contexts we mentioned in this section clearly show how the intuitive system produces strong emotional judgments that are powerful enough to constrain the functioning of the deliberative system.

## How Does Cognition Regulate Emotion?

In the previous discussion, we delineated contexts in which emotion is likely to override reason to influence our behavior choices. However, intuitive responses, if unchecked, may turn out to be suboptimal and even destructive. Cognition can exert powerful modulation on emotion in many circumstances. Emotion regulation is a set of controlled processes that explicitly involve strategies to initiate, increase, maintain, or decrease the occurrence, intensity, or quality of experienced feeling states (Eisenberg et al., 2000; Gyurak et al., 2011; Webb et al., 2012). In contrast with the aforementioned definition of emotion regulation, some other research suggests that emotions are self-regulating implicitly. Emotions are not just regulated by other cognitive processes, but more importantly, emotions regulate themselves via learning and experience (Kappas, 2011a,b). To address the divergent results of earlier studies, Gyurak et al. (2011) provided a dual-process framework to integrate the research on explicit emotion regulation and implicit emotion regulation. According to Gyurak et al. (2011), explicit emotion regulation is made up of a set of processes that involve conscious effort for initiation and monitoring when implemented. Individuals are aware of what they are currently doing. Implicit emotion regulation is evoked automatically by the emotional stimulus itself, and it can occur without awareness. In this review, we focus more on explicit emotion regulation during which conscious effort modulates emotion. Notably, the most widely used model is the process model of emotion regulation (Gross, 1998). This model differentiates three major strategies as attentional deployment, cognitive change, and response modulation (D’Zurilla et al., 2003; Webb et al., 2012). In this section, we highlight how cognition modulates emotion and how these two processes cooperate to affect ongoing behavior.

### Attentional Deployment

Attentional deployment includes two aspects, namely, distraction and concentration (Webb et al., 2012). Distraction means moving attention away from the current situation or directing attention to a different aspect of the situation (Gross and Thompson, 2007). This has been shown by instructing individuals to think of something else or to perform an unemotional task (Masuda et al., 2010). Concentration can be shown when participants are directed to focus on or make judgment about their emotional experience. One widely used concentration strategy is affect labeling (Hariri et al., 2000; Lieberman et al., 2007, 2011; Ramirez and Beilock, 2011). Typically, in affect labeling studies, participants are asked to choose either from a pair of pictures or a pair of words. The pictures or words are related to the emotional content of a target picture (Lieberman et al., 2011). A recent empirical study (Ramirez and Beilock, 2011) applied affect labeling in a school setting and found that writing about testing worries boosted exam performance in the classroom. It is likely that writing attenuates the burden that worries place on working memory, thus providing individuals an opportunity to reevaluate the anxious and stressful expression in a manner that reduces the necessity to worry altogether (Klein and Boals, 2001; Ramirez and Beilock, 2011).

Some neuroimaging studies show decreasing amygdala activity combined with an increasing lateral PFC when individuals attempt to evaluate emotion-related stimuli (Hariri et al., 2000, 2003). It is possible that judgment or evaluation itself might engage a greater attention load, limiting the process of perceptual input and as a result constraining amygdala activation (Bishop et al., 2004). If this is the potential mechanism of attentional deployment’s effects on decision making, we assume that cognition and emotion engage common limited resources because paying less attention to the emotional stimuli modulates the processes of both cognitive evaluation (e.g., increased PFC activation) and emotional appraisal (e.g., reduced amygdala activation). Emotion and cognition travel opposite directions in attention manipulation, outlining a reciprocal PFC-amygdala relationship with one domain being strengthened and the other one weakened.

### Cognitive Change

Cognitive change strategy, also termed reappraisal, refers to efforts that change the way individuals appraise the situation and alter its emotional significance (Aldao et al., 2010). Participants can modulate their current emotion, for example, by providing a less negative interpretation when they meet an undesirable picture content (Hajcak and Nieuwenhuis, 2006) or increasing the sense of objective distance by viewing picture content from a third-party perspective (Ochsner et al., 2004b). In general, data from fMRI studies have found that reappraisal of negative stimuli activates the dorsal PFC system, which facilitates the selection and application of cognitive change strategies and attenuates activity in the emotional system including the amygdala and insula (Ochsner et al., 2002; Phan et al., 2005; Buhle et al., 2014). We speculate that the PFC system modulates the amygdala response toward emotional stimuli, showing an interaction between emotion and cognition in stimuli reinterpretation. Notably, cognitive change does influence the reasoning process (cognitive process) beyond simply removing the emotion, extending its influence to change reward encoding and motivation processes (Staudinger et al., 2009, 2011; Martin et al., 2013). For example, in a behavioral economics study (Martin et al., 2013), participants engaged in two reappraisal strategies with different goals—to increase or decrease the importance of a coming decision. The results showed that emphasizing the perceived significance of the next decision decreased risk taking, whereas reducing the perceived importance of the coming decision increased the risk taking. However, there was no emotion arousal change when individuals engaged in reappraisal with opposite goals. These results suggest that cognitive change can flexibly alter reward encoding and economic behavior with regulation goals. Another neuroimaging study (Staudinger et al., 2011) revealed that reappraisal would modulate reward cue and motivation processing with increases of activity in the dorsolateral PFC and attenuation of anticipatory reward cue encoding in the putamen. The researchers suggested that emotion regulation areas such as dorsolateral PFC might exert modulatory control over the putamen to bring about such effects.

To recap the aforementioned studies, it seems that cognitive change results in amplified activation in the cognitive system (e.g., dorsolateral PFC), and reduced activation in the appraisal system (e.g., amygdala and insula). But this is not always the case. When anticipating responses that precede an upcoming emotional event, research also observes increasing, rather than decreasing, activation in emotional regions such as amygdala and insula (Ochsner et al., 2004a). The distinct directional nature of these observed interactions is still unclear. One possibility is that in regulating an existing or ongoing emotional response, the PFC system exerts a top-down inhibitory effect on amygdala response (Banks et al., 2007); whereas in anticipating how a stimulus might feel, strengthened amygdala-frontal coupling reflects an enhanced cognitive effect due to a failure to down-regulate amygdala activity. Another possibility is that using cognitive change to regulate an existing emotion and to anticipate an upcoming emotional response might involve two distinct neural modes. Although it remains unclear why cognitive change leads to distinct activation in the amygdala, it is clear that cognitive change depends on the interaction of a prefrontal system supporting cognitive process and a subcortical system engaging emotional information processing.

### Response Modulation

Response modulation means enhancing or suppressing one’s subjective feelings, behavioral responses, or physiological responses within an emotion-eliciting situation (Gross and Barrett, 2011; Webb et al., 2012). Researchers have manipulated response modulation in different ways (Webb et al., 2012). For example, Schmeichel et al. (2006) instructed subjects to view a disgust-eliciting film clip and exaggerate their reactions (enhance the expression of emotion); Gross and Levenson (1993) asked participants to watch a film clip, but to hide their emotion and act as if they did not feel anything at all (suppress the expression of emotion); Quartana and Burns (2007) instructed participants to suppress all of their feelings and try to push all their emotion out of their mind (suppress the experience of emotion); and Dalgleish and Yiend (2006) asked participants to inhibit thinking about a personal distressing event that had been described before (suppress thoughts of the emotion-eliciting event). Indeed, relative to emotion enhancement (exaggerate emotional reaction), emotion suppression (suppress expression and experience of emotion) is more widely explored. Many empirical studies have investigated the function of emotion suppression in health, well-being and social interaction (McCaul et al., 1979; Anderson and Green, 2001; Nørby et al., 2010). Evidence suggests that emotion suppression can slow down heart rate and breathing (McCaul et al., 1979) and help us to forget unwanted memories (Anderson and Green, 2001), but it may also increase blood pressure (Burns et al., 2007) and even lower individuals’ social support and satisfaction (Srivastava et al., 2009).

Recent neuroimaging research has begun to examine the neural bases of emotion suppression (Davidson et al., 2000; Lévesque et al., 2003; Goldin et al., 2008). Consistent evidence suggests that suppression increases activation of the prefrontal regions such as the ventrolateral PFC, the dorsolateral PFC and the OFC (Davidson et al., 2000; Beauregard et al., 2001; Lévesque et al., 2003; Goldin et al., 2008). This increased response correlates with a simultaneous decreased response in limbic regions such as the amygdala and hypothalamus (Davidson et al., 2000; Beauregard et al., 2001). The PFC, especially the dorsolateral PFC, is implicated in executive process referring to the ability to control information processing. The amygdala is implicated in the evaluation of emotional significance of stimuli (Lane, 2000) and the hypothalamus is associated with expressing emotion (Carter, 1998). These results seem to outline neural circuitry underlying suppression of emotion, suggesting that the prefrontal regions constrain the evaluation and expression of emotion during the suppression task. In fact, it remains unclear whether the underlying mechanisms by which prefrontal regions regulate emotion evaluation and expression extend to the decision domain. Few studies have investigated how suppression affects our decision making. In a recent behavioral study, Heilman et al. (2010) found that suppression of positive emotion (but not negative emotion) could reduce individuals risk aversion. Building on Heilman et al.’s (2010) results, Panno et al. (2013) found that the habitual use of a suppression strategy (a naturally occurring individual difference in emotion regulation) was associated with decreased risk taking. The authors suggested that suppression induced less risky choices due to participants paying more attention to avoiding negative emotion elicited by negative potential outcomes. However, in this study, the relation of suppression and decision making is just correlational and comes from self-report. Thus, it is not clear how suppression itself influences decision making and what are the neural bases of suppression and decision making. Whether our assumption that prefrontal regions constrain the evaluation and expression of emotion during the suppression task can also extend to the decision domain awaits further investigation.

### Critical Summary

In this section, we demonstrated that through emotion regulation strategies including attentional deployment, cognitive change and response modulation, cognition, as relative to emotion, may play a stronger role in judgment and decision making. More importantly, we also point out that the ability to regulate emotion depends upon the interaction between the PFC supporting cognitive control and a subcortical system that represents different types of emotion-related information. More specifically, subcortical regions facilitate processing of emotional information, and prefrontal regions can constrain the evaluation and expression of emotion during emotion regulation tasks. When concentrating on (attention deployment), reappraising (cognitive change), and suppressing (response modulation) emotion stimuli, cognition exerts a top-down regulation of the amygdala responses. The integration of passion and reason in decision making can be strongly confirmed by lesion studies from animals (Málková et al., 1997; Schoenbaum et al., 2003) and human beings (Hampton et al., 2007). For example, human subjects with amygdala lesions displayed a profound change in PFC activity related to reward expectation and behavioral choice, indicating that production of signals related to behavioral choice in PFC relies directly on input from the amygdala (Hampton et al., 2007). Thus, based on the aforementioned studies and other research confirming that emotion and cognition are integrated to determine ongoing behaviors (Phelps, 2006; Pessoa, 2008; Pessoa et al., 2012), it is reasonable to predict that emotion regulation depends on the interaction of a prefrontal system supporting cognitive process and a subcortical system engaging emotional information processing.

## The Interactive Influence Model of Emotion and Cognition

The most influential arguments to account for the antagonism of emotion and reason in decision making come from “dual-process models” (Evans, 2008; Keren and Schul, 2009; Kahneman, 2011). The two processes are assumed to be engaged in distinct mental modes—one mental system that is emotional and intuitive, and another system that is rational and deliberative. According to the implication of dual-process models, intuitive process can produces an emotional judgment, and deliberative process may go against the intuitive initial reaction. Though the dual-process models can explain the antagonism of emotion and reason, there remain several limitations. For one, the idea of dual processes is conceptually unclear and oversimplifying (Kruglanski et al., 2006; Keren and Schul, 2009). It seems that nearly all dual-process models embrace the dichotomy of intuition (emotion) and deliberation (reason), yet, whether there are two systems rather than one or more systems in mental processes remain unclear (Pessoa, 2014). For another, the dual processes fail to account for how emotion and reason interplay to influence our decisions in daily judgments. Take one example of the interaction of reason and intuition: a person may reason their way to a moral view (such as that verbal abuse is wrong), and this moral view might change to be intuitive overtime (Pizarro and Bloom, 2003). Take another example: individuals implementing emotion regulation show simultaneously increasing brain activity in the PFC, which supports cognitive process, and decreasing activity in limbic system, which is implicated in emotion process (Heilman et al., 2010; Sokol-Hessner et al., 2013). The above examples suggest that the mechanism by which a reasoned choice develops into an intuitive one, and the mechanism by which emotion regulation strategies engage the interconnection of cognitive process and emotional process, are not well elaborated in dual-process models.

**FIGURE 3:**
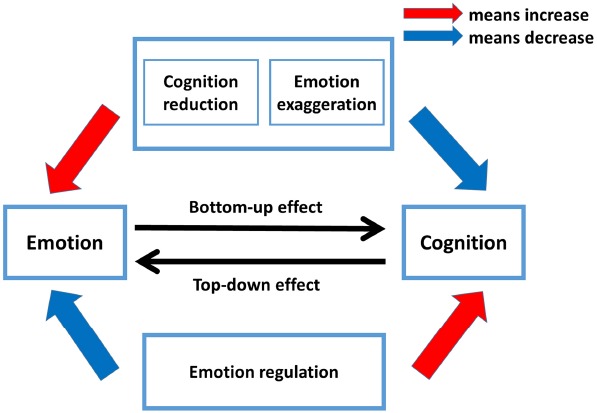
**The interactive influence model of emotion and cognition (IIEC).** Decision making depends upon the interaction of emotion and cognition. Two typical contexts, namely cognition reduction (cognitive capacity is reduced) and emotion exaggeration (emotional reaction is enhanced), increase the power of emotion but decrease the power of cognition. The cognition reduction can be seen in decision setting such as ambiguity, time constraint, and ego depletion. The emotion exaggeration can be observed in decision settings such as proximity, social distance, and social instinct. In these two contexts, emotion can overwhelm cognition and exert a bottom-up influence on decision making. Cognition can modulate emotion reaction by consciously using emotion regulation strategies such as attention deployment, cognitive change, and response modulation. These emotion regulation strategies help to strengthen the capacity of cognition and constraint the power of emotion. With emotion regulation, cognition can exert a top-down effect during decision making.

In this review, drawing on findings mentioned in previous sections, we integrate the antagonism and integration of passion and reason in decision making within a framework, labeled “The interactive influence model of emotion and cognition (IIEC)” (see Figure 3). The IIEC model differs from previous accounts of how emotion (or cognition) overrides cognition (or emotion) as well as how they cooperate with each other to affect decision making. In this model, we postulate that (1) emotion transcends cognition to affect decision making by reducing cognitive capacity (cognition reduction) and enhancing emotional response (emotion exaggeration); (2) cognition overwhelms emotion to influence decision making by explicitly implementing cognitive control via emotion regulation strategies; (3) decision making depends upon the interaction of emotion and cognition.

More specifically, emotion exerts a bottom-up effect under two typical contexts—cognition reduction and emotion exaggeration. The cognition reduction context can be observed in decision settings where information is incomplete (ambiguity), decision time is limited (time constraint), and self-regulation is impaired (ego depletion). The emotion exaggeration can be seen when individuals meet threatening cues or immediate reward (proximity), self-related information (social distance), and social stimuli (social instinct). In these two contexts (cognition reduction and emotion exaggeration), the power of emotion is potentiated and the capacity of cognition is diminished, thus leading emotion to override cognition to affect decision making. As for cognition, it can exert a top-down effect through the conscious use of emotion regulation strategies. When concentrating on (attention deployment), reappraising (cognitive change), and suppressing (response modulation) emotional stimuli, cognition is able to alter emotion’s evaluation and expression. That is, using emotion regulation strategies strengthens the capacity of cognition and reduces the power of emotion. Neither emotion nor cognition alone guarantees a sound decision making. This is because decision making depends upon the interaction of a subcortical system (e.g., amygdala, hypothalamus, thalamus, putamen, and hippocampus) engaging in emotional information processing and a prefrontal system (e.g., dorsal PFC, ventromedial PFC, dorsomedial PFC, OFC) supporting cognitive process. Such interaction is embedded at the perceptual and executive levels. Subcortical system components such as the amygdala are involved in processing of perceptual input (Bishop et al., 2004) that helps to generate and evaluate emotions such as anger, fear or disgust, while the prefrontal system exerts modulatory control on representation of perceptual stimuli (Buhle et al., 2014), and thus in turn changes the expression and evaluation of emotion.

The IIEC model can be supported by evidence from numerous behavioral studies, neuroimaging research, and lesion cases. First, data from research on impulsive behaviors indicate that emotion exerts a bottom-up effect on cognition. For example, healthy participants are more impulsive with an immediate reward than a delayed one (Frederick et al., 2002; Green and Myerson, 2004), and individuals with drug addiction are over-responsive to drug-related cues, showing increasing activity in the amygdala and other parts of the limbic system (Koob and Le Moal, 2007). It is possible that these affective signals (immediate reward and drug-related cues), exaggerate emotional responses, and consequently exert a bottom-up influence on the cognitive system as the IIEC model posits.

Second, research focusing on emotion regulation during decision making suggests that the prefrontal system exerts a top-down effect on emotion, outlining the integration of emotion and cognition in decision making (Sokol-Hessner et al., 2009, 2013; van’t Wout et al., 2010; Miu and Crişan, 2011; Grecucci et al., 2013; Panno et al., 2013). For instance, the use of emotion regulation techniques (e.g., reappraisal) in behavioral economics and neuroeconomics studies suggests that emotion regulation helps to reduce loss aversion—a phenomenon in which individuals tend to strongly prefer avoiding losses to acquiring gains (Kahneman and Tversky, 1984). Moreover, emotion regulation also increases PFC responses accompanied by decreasing amygdala responses to losses (Sokol-Hessner et al., 2013). According to the IIEC model, it is likely that emotion regulation involves the use of cognitive control (reappraisal or suppression) that modulates representations of emotional stimuli and consequently attenuates activity in the amygdala.

Third, research on decisions about altruism, trust, and fairness shows the interaction of emotion and reason during social interaction (Moll et al., 2006; Spitzer et al., 2007; Buckholtz et al., 2008; Rilling and Sanfey, 2011; FeldmanHall et al., 2015). Charitable donation is a difficult social decision involving psychological conflict between self-interest and the interests of others (Rilling and Sanfey, 2011). In one study (Moll et al., 2006), researchers found that voluntarily donating money to a specific charity activated the mesolimbic dopamine system such as components of the ventral tegmental and the ventral striatum. The mesolimbic reward system engaged by donation was engaged in the same way when individuals accepted monetary reward, suggesting that donation and earning money shared a similar system of reward reinforcement (O’Doherty et al., 2006). Remarkably, the PFC was also recruited when individuals decided whether to donate or not, indicating that individuals exerted cognitive control over selfish material interests. The brain processes involved in donation decisions are analogous to brain processes involved in other costly altruistic decisions during which participants experience feelings of other-oriented empathy (FeldmanHall et al., 2015) or obtain more money than their partners in economic games (Yu et al., 2014). These results, according to the IIEC model, might suggest that the perceived joy (emotion) of donation (or helping others) facilitates exertion of cognitive control (cognition) over self-interests and thus guarantees prosocial behaviors. Indeed, not only social reward, but also punishment threat motivates participants to behave prosocially. When punishment is available, individuals’ increases in norm compliance are positively correlated with activation in the caudate and the dorsolateral PFC (Spitzer et al., 2007). The caudate is involved in processing information about positive or negative reinforcers (Delgado et al., 2003; Spitzer et al., 2007). These results might indicate that the perceived threat (caudate) motivates individuals to override proponent impulses (dorsolateral PFC), therefore resulting in more fair decisions. Taken together, humans make altruistic and fair decisions based on the interaction of emotion and cognition—the perceived reward or punishment motivating individuals to override self-interested impulses.

Fourth, lesion studies further indicate how emotion and cognition cooperate to affect decision making (Davidson et al., 2000; Shiv et al., 2005; Clark et al., 2008). For example, as we mentioned earlier, humans with amygdala lesions exhibited a profound change in PFC activity related to reward expectation and behavioral choice, suggesting that production of signals related to behavioral choice in PFC relies directly on input from the amygdala (Hampton et al., 2007). Similarly, other research also documents that amygdala damage abolishes monetary loss aversion (De Martino et al., 2010). These results, according to the IIEC model, may suggest that deficits of amygdala constrain processing of perceptual stimuli, lower individuals’ sensitive to loss or reward, and as a result interfere with reason processes that calculate the losses and gains based on existing information. Besides the amygdala, another prefrontal region, ventromedial PFC, is also well documented in decision making (Berlin et al., 2004). The Somatic Marker Hypothesis (Damasio, 2008) suggests that bodily states that were previously associated with choice alternatives are retrieved by the ventromedial PFC. Bodily states, according to Somatic Marker Hypothesis, can transform into emotions. Therefore, this hypothesis implies that ventromedial PFC connects emotion and cognition during decision making. Patients with ventromedial PFC damage increase betting and impulsive behaviors (Bechara et al., 2000; Berlin et al., 2004; Clark et al., 2008), and they exhibit more subjective anger but less subjective happiness than normal participants (Berlin et al., 2004). The IIEC model proposes that decision making depends upon the interaction of emotion and cognition, so it is reasonable to infer that dysfunction of ventromedial PFC disrupts the connection of emotion and cognition. Therefore, the prefrontal system fails to control the process of emotion, which makes emotional stimuli salient and in turn exaggerates emotional responses leading to emotion-dominated behaviors.

To sum up, the IIEC model we posit serves as a launching point for understanding the interplay of emotion and reason in decision making. First, the framework we established can be applied to understanding a diverse range of human decision making behaviors in which emotion plays a prominent role. We shift the question from “whether emotions affect decision making” to “when and how does emotion affect decision making.” Our model suggests that when cognition capability is reduced or emotion reaction is enhanced, emotion can overwhelm reason to exert a bottom-up effect during decision making. Second, this model broadens our understanding on how cognition affects decision making by modulating emotional processes. Through the conscious use of emotion regulation strategies such as attention deployment, cognition change, and response modulation, cognition can modulate the expression and evaluation of emotion during decision making. Third, the IIEC model can also be applied to understanding the decision-making impairments that are associated with brain damages. The IIEC model suggests that decision making depends upon the interaction of emotion implicated in subcortical cortex and reason implicated in PFC. Damage in either prefrontal or subcortical regions leads to maladaptive behaviors and impaired decisions. Besides the contributions of IIEC model, two main limitations should be noted. For one, we put forth a framework about when emotion plays a prominent role in decision making and when reason does, but we fail to articulate the baseline balance between emotion and cognition during decision making. Kahneman and Tversky (1979) suggested that individuals usually use automatic processes to generate judgments and attitudes and then use controlled processes to make necessary adjustments; that is, emotion process may initiate some default action tendency and the reason process evaluates and modulates such tendency according to current goals and social interactions. If this is the case, we might assume that the default action tendency generated by emotion is the baseline of the interaction of passion and reason. Without this baseline, cognition might fail to exert cognitive control. However, this assumption has not been clearly elaborated. For another, we have applied this model to explain some research in the decision domain; however, whether this model can also apply to other domains remains unclear. A recent study conducted by Pessoa et al. (2012) showed that low-threat stimuli (fearful and happy face) improved response inhibition but high-threat stimuli (stimuli previously paired with mild shock) impaired performance. According to the IIEC model, we might suggest that high-threat stimuli exaggerate the emotional response, which strengthens the power of emotion and then interferes with the executive control system, leading to poor performance. However, this is just one example, and more research is needed to test our hypothesis.

## Concluding Remarks

The conceptual framework model we have put forth—IIEC—posits that emotion and reason cooperate to shape our decision making. In this model, we suggest that decision making depends upon the interaction of a subcortical system engaging emotional information processing and a prefrontal system supporting cognitive process. We emphasize that emotion plays a more important role when cognitive capacity is reduced (cognition reduction) or when emotion is greatly strengthened (emotion exaggeration) by emotional stimuli or cognitive tasks. We also highlight that cognition can regulate emotion through strategies including attentional deployment, cognitive change, and response modulation. This model is important because it takes into account both antagonism and integration of emotion and reason in a more dynamic framework as contrast to previous dual-process models which embrace the competition of passion and reason. This model re-interprets evidence that until now was used to support the dual-process models, suggesting that decision making relies on the interaction of emotion and cognition and that whether emotion or cognition makes a more prominent role depends on specific decision contexts.

## Author Contributions

JL wrote the first draft of the paper. RY provided critical revisions. Both authors reviewed the manuscript.

### Conflict of Interest Statement

The authors declare that the research was conducted in the absence of any commercial or financial relationships that could be construed as a potential conflict of interest.
