# Erratum on: Follow the heart or the head? The interactive influence model of emotion and cognition

**DOI:** 10.3389/fpsyg.2015.01039

**Published:** 2015-07-10

**Authors:** Frontiers Production Office

**Affiliations:** Frontiers Production Office, FrontiersLausanne, Switzerland

**Keywords:** emotion, reason, dual-process, decision making, emotion regulation

Reason for erratum:

Due to a misunderstanding the name of the corresponding author was published as Jiayi Luo instead of Rongjun Yu. The contact information is correct as originally published.

This error does not change the scientific conclusions of the article in any way. The publisher apologizes for this error. The original article has been updated.

